# The effect of dual-task training on cognition of people with different clinical conditions: An overview of systematic reviews

**DOI:** 10.1016/j.ibror.2020.06.005

**Published:** 2020-07-01

**Authors:** Henrique Nunes Pereira Oliva, Frederico Sander Mansur Machado, Vinícius Dias Rodrigues, Luana Lemos Leão, Renato Sobral Monteiro-Júnior

**Affiliations:** a Centro Universitario FIPMoc (UNIFIPMoc), Montes Claros, MG, Brazil; b Post-Graduate Program of Health Sciences (PPGCS), State University of Montes Claros (UNIMONTES), Montes Claros, MG, Brazil; c Grupo de Estudos em Neurociência, Exercício, Saúde e Esporte (GENESEs) of UNIMONTES, Montes Claros, MG, Brazil; d Departamento de Educação Física e Desporto da UNIMONTES, Montes Claros, MG, Brazil; e Post-Graduate Program of Medicine (Neurology/Neuroscience), Federal Fluminense University, Niteroi, RJ, Brazil; f Neuroscience of Exercise Institute, Aroldo Tourinho Hospital, Montes Claros, MG, Brazil

**Keywords:** Dual-task training, Dual-task intervention, Motor cognitive training, Cognition, Executive functions, Memory, Decision making, Spatial navigation

## Abstract

**Background:**

The number of patients with cognitive impairment increases as the population becomes older. This perspective may persist a burden on health care systems unless considered new options of prevention and treatment. The aim of this meta-synthesis is to analyze different systematic reviews on the effectiveness of dual-task training (DTT) on cognition and motor function of different people.

**Methods:**

A systematic search of systematic reviews published until October 2019 was conducted in PubMed/Medline, Scopus and Cochrane databases addressing studies which investigated the effect of DTT compared to control or other intervention on cognitive functions of healthy or unhealthy individuals. Three steps were followed to retrieve studies: reading title, abstract and full text. Checklist Assessing the Methodological Quality of Systematic Reviews (AMSTAR) was used to assess the quality of selected articles.

**Results:**

In terms of quality of evidence, according to AMSTAR, 62.5 % of the reviews were rated as being “low” and 37.5 % were graded as “moderate” quality. Two main themes were identified among the studies’ outcomes: Improvement on mobility performance or postural stability; and beneficial effect on cognitive function. In terms of effect size, there were reported an important variation, having more significant results for findings involving mobility and modest effect for findings regarding cognitive function.

**Conclusion:**

People with different clinical conditions could benefit from dual-task training. The benefits may encompass general cognitive functions, memory, physical performance, gait and balance, to name a few aspects.

## 1 Introduction

Cognitive impairments such as dementia and others are diagnosed after discerning clinician’s assessment of significant cognitive changes. It is important to highlight that there is an expected degree of cognitive slowing due to normal aging and it differs from conditions that imply compromise to social/occupational functioning, i.e., cognitive impairment (Hugo, Ganguli, 2014; Alzheimer’s Association, 2018). When it comes to the implications of cognitive impairments it has multiple consequences going beyond the individual affected. There is an impact on its family, the economy and the health system, to name a few. It is also concerning because this problem is often associated with elderly, which is the population’s age group expected to grow as a country becomes more developed and with a higher income (Hugo, Ganguli, 2014; Matthews et al., 2013).

There is reported an estimated number of 115.4 million people worldwide to be living with dementia by 2050, whereas there were reported 35.6 million in 2010 (Prince et al., 2013). Most dementias are not curable, so they affect prevalence of major cognitive impairment with higher prevalence in groups with longer life expectancy. Cognitive impairment increases with increasing age. It is reported a higher prevalence in women, when compared to men, probably because women live longer (Hugo, Ganguli, 2014; Plassman et al., 2007). In developed countries the prevalence of this disease is in 5–10 % of people with 65+ years old. Dementia has been more associated with Latin America population and less with sub-Saharan Africa. However mild cognitive impairment (MCI) that has not yet evolved to dementia is poorly reported due to the difficulty of definition and differentiation of subtypes in studies (Ward et al., 2012; Hall et al., 2009).

Different types of studies address the association of older age with a wide range of physical and mental problems. One important problem is related to decline in motor function, showing that older people have greater risk of falls. The more sedentary the greater the risk of falls, related to regression of balance and motor performance overall (Norouzi et al., 2019). Motor function in the elderly is a topic of constant importance since it is a modifiable risk factor for falls and falls are related to a great morbidity and mortality in this population (Guirguis-Blake et al., 2018).

There are several limitations of studies on costs of cognitive impairments. Some explanations for that are in lack of longitudinal data or unprecise and shortage in notification and registration of cases related to its specific costs (Knapp et al., 2013; Zhu et al., 2013; Mauskopf et al., 2010). In a study conducted in United States with over one hundred elderly, comparing costs of the ones with cognitive impairment with control group, it was found higher costs in caring of the first ones. The larger cost was reported to hospitalizations. Also, the study showed that annual direct medical cost and informal care use per person with MCI was more than two times higher than for those without MCI (Zhu et al., 2013; Langa et al., 2001; Barnes e Yaffe, 2011). Moreover, dementia could correlate or evolve from a condition of MCI, and studies suggest that dementia represent as a substantial financial burden on society as heart diseases and cancer (Hurd et al., 2013).

Research has explored and supported the view of positive effects of exercise-based interventions (acute and long-term effects) on cognitive functions and motor functions in older people (Norouzi et al., 2019); The type of exercise has varied from aerobic exercise to resistance training and dual-task training (DTT). Also, according to past studies, DTT appear to provide more consistent cognitive or motor function benefits in older adults, when compared to the other exercise interventions. There are different types of DTT: for example, DTT with two motor assignments like strength training and balance training exercised simultaneously (called motor-motor DTT) and motor-cognitive DTT, having cognitive task associated with resistance training (called motor-cognitive DTT). Dual-task training (DTT), such as the combination of cognitive training and motor training, have shown improvement in memory, balance and mobility in older people (Norouzi et al., 2019; Brustio et al., 2018).

Patients with several different impairment conditions could benefit from DTT and, as an example of that, studies conducted with elderly with chronic stroke showed improvement in mobility and balance (Tetik Aydoğdu et al., 2018; Pang et al., 2018). Besides the perspective on treatment, DTT is important to prevent cognitive decline, as showed in studies with older adults at risk for future cognitive decline (Gregory et al., 2017). According to Ferreira et al. (2019) the ability to perform two tasks simultaneously seems to be compromised in patients with Alzheimer’s disease, and unaltered for individuals with Major Depression. Also, in cases of dementia, DTT was proven effective in improving specifically trained dual-task performance in patients with the condition (Lemke et al., 2019).

There are no studies analyzing the minimal and maximal effect of DTT on cognition observed in different people with different clinical conditions. Moreover, according to researchers like He et al. (2018), there is still scarce evidence to support the use of DTT to enhance dual-task walking, balance, and cognitive function. Hence, despite the perspective of improvement in multiple functions for patients subjected to DTT clinical significance of the treatment effect is still uncertain (Plummer and Iyigün, 2018). Therefore, the aim of this meta-synthesis is to analyze systematic reviews which have investigated the effect of DTT on cognitive functions, postural stability and mobility of people with different clinical conditions.

## 2 Methods

### 2.1 Design, Information sources and search strategy

The study consists in an overview (meta-synthesis) of systematic reviews. PICO strategy was applied to retrieve systematic reviews published until October 2019, addressing studies which investigated healthy or unhealthy individuals (Population) and the effect of DTT (Intervention) compared to control (Comparator) on cognitive functions, stability and mobility (Outcomes). According AMSTAR, two databases are required at least in a systematic review. Searches were handled at PubMed/Medline, Scopus, and Cochrane databases using the following search terms filtered on title or abstract, combining Boolean operators OR/AND: "dual-task training" OR "dual-task intervention" OR "dual-task activity" OR "dual-task exercise" OR "motor cognitive training" AND “cognition” OR "cognitive functions" OR "executive functions" OR “memory” OR "decision making" OR “reasoning” OR "inhibitory control" OR "cognitive flexibility" OR "spatial navigation". The search was conducted at 31 st October 2019, and retrieved the published systematic reviews from 2009 up to date. The terms were retrieved by title/abstract in each database. When the database did not provide this option, it was retrieved by abstract. Also, “Systematic Review” or “Review” was used as filter term when database displayed this option. Independently of the language, articles have at least their abstracts indexed in English in these databases. Therefore, English language was a requisite related to the search strategy. One author performed the literature search. Data extraction from chosen records was processed by two researchers independently, one of which performed the creation and insertion of data in the table. Disagreement related to the inclusion and screening process of a given result was decided by another author, as supervisor.

Grey literature search was considered in order to minimize the publication bias. Authors reviewed potential articles in the list of references of each systematic review included in the present work. In addition, it was searched non-published reviews on NIHR Centre for Reviews and Dissemination. However, the material retrieved was not included given the consensus among authors, based on lack of detail and peer review.

### 2.2 Eligibility criteria and data analysis

Systematic reviews with or without meta-analyses with clear qualitative or quantitative data were selected. Three steps were followed to retrieve studies: 1) reading title, 2) reading abstract and 3) reading full text. Information regarding any cognitive outcome was extracted. Checklist "Assessing the Methodological Quality of Systematic Reviews (AMSTAR)" was used to assess the quality of selected articles (Shea et al., 2007). The sixteen multiple choice questions related to the AMSTAR tool checklist (https://amstar.ca/Amstar_Checklist.php) were responded for each study included (Shea et al., 2017). Two examiners went through the process, independently, using ‘no’, ‘partial yes’, ‘yes’ or ‘not applicable’, in given cases. The following quantitative data was considered: Overall effect size, confidence interval, and heterogeneity. A classification of overall effect size was done according with Cohen’s d effect size (Cohen, 2013) as follow: >0.30 small effect, between 0 and 0.50 medium effect, and >0.80 large effect.

## 3 Results

### 3.1 Search outcome

**Fig. 1:**
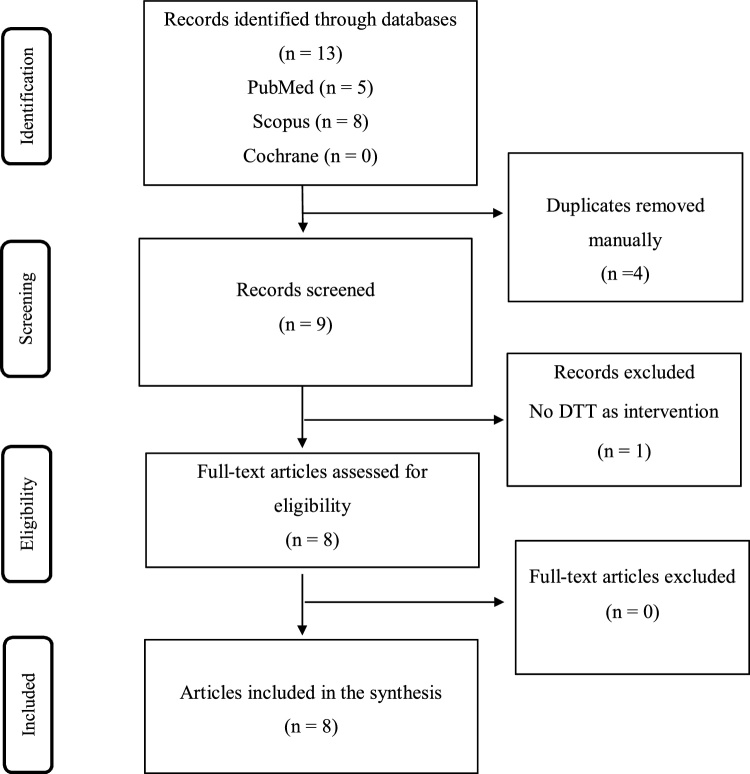
Study selection flow chart.

Systematic Reviews were selected if they clearly investigated DTT on any cognitive, stability or mobility outcome of healthy or unhealthy persons. Persons (healthy or unhealthy individuals), Intervention (DTT), Comparators (control or other intervention group), and Outcomes (cognitive functions, stability and mobility) were inspected and selected if all items were confirmed, addressing PICO strategy. Eight systematic reviews among 13 identified in databases were analyzed (Fig. 1).

The search strategy identified 13 potentially eligible systematic reviews from all electronic databases. After manual removal of duplicate articles and screening of titles and abstracts, 8 full-text publications were comprehensively evaluated and selected for the present review (Agmon et al., 2014; Fritz et al., 2015; He et al., 2018; Lauenroth et al., 2016; Law et al., 2014; Pichierri et al., 2011; Plummer and Iyigün, 2018; Wajda et al., 2017). One systematic review with meta-analysis was identified, Plummer and Iyigün (2018). The characteristics and main findings of the reviews included are presented in Table 1.

**Table 1:** Characteristics and main findings of the reviews included: sample, number of studies, number of subjects, study designs, intervention, outcomes and results.

Author, year	Sample characteristics	Number of studies included	N (total)	Studies Design	Intervention	Outcomes	Results
Agmon et al., 2014	Aged 60 years or older, healthy adults.	22	709	RCT (16), Controlled and Uncontrolled pretest to posttest (4), Case control (1) and Case series (1)	Interventions required a minimum of 180 min of training over at least 3 total days.	Dual-task postural control was measured as an outcome.	18 demonstrated improvement in some aspect of dual-task performance and 4 did not; From those, 3 demonstrated improvement for postural control and the concurrent cognitive/motor task. 7 improved in one but not both. The 8 left did not measure dual-task.
Fritz et al., 2015	Adults with brain injury, PD and AD	14	296	RCT (3) and RMD (11)	Intervention protocols included cued walking, cognitive tasks paired with gait, balance, and strength training and virtual reality or gaming.	Mobility, single- and dual-task gait velocity and stride length or balance, as well as cognitive performance as outcome of interest.	DTT improved single-task gait velocity and stride length in subjects with PD and AD, dual-task gait velocity and stride length in subjects with PD, AD, and brain injury, and may improve balance and cognition for PD and AD.
He et al., 2018	Stroke survivors (12 studies chronic stroke, one study with sub-acute stroke).	13	457	RCT	Experimental group received DTT.	Mobility and balance performance in single-task or dual-task condition. Also, measurements that reflected the participants’ cognitive function, and/or ability to perform, and/or participation level.	DTT induced improvement in single-task walking function. Cognitive-motor balance training improved single-task balance function. Beneficial effect of DTT on cognitive function was provided by one study only.
Lauenroth et al., 2016	Adults; cognitively healthy participants (14 studies). People with MCI (2 studies). Patients with stroke pathology (1 study). Patients with dementia or AD (3 studies).	20	1145	RCT (15) and CT (5).	Combined intervention with a stimulating physical training, as well as cognitive training, conducted either simultaneously in the form of DTT or subsequent training interventions.	Cognitive outcomes as a study’s endpoint. influence of combined physical and cognitive training on cognition.	Suggest that a training scheme of 1–3 hours weekly for 12–16 weeks (or more) is more likely to lead to detectable improvements in cognitive performance than other training schemes.
Law et al., 2014	Aged 60 and older with or without cognitive impairment or dementia but no mental or neurological disorders.	8	473	RCT (4) and NRCT (4)	Combined cognitive and exercise training.	Cognitive functions assessed using neuropsychological tests as primary or secondary outcomes.	Cognitively healthy populations, as well as with MCI, AD and other dementia showed significant benefits of combined cognitive and exercise interventions on general cognitive functions, memory, and functional status.
Pichierri et al., 2011	Adults with history of falls, balance disorders, MCI or osteoporosis. Also, patients after stroke and traumatic brain injury.	28	1564	RCT (16), NRCT (1), Case study (5), Case-control (1), two groups control (1) and Pre-Post (4).	Isolated cognitive rehabilitation intervention (3 studies), dual-task intervention (7) and applied a computerized intervention (19).	Cognitive and cognitive-motor interventions affecting physical functioning.	The evidence on the effectiveness of cognitive or motor-cognitive interventions to improve physical functioning was found to be limited. However, most studies showed these interventions can enhance physical functioning.
Plummer and Iyigün, 2018	Individuals with stroke.	7	125	RCT (5), Case series (1) and uncontrolled studies (1).	Dual-task gait training with cognitive-motor paradigm and motor-motor paradigm.	Dual-task gait speed.	Exercise and gait training interventions, especially DTT may improve dual-task gait speed after stroke, but the clinical significance was unclear. Current effect size estimates lack precision due to small sample sizes of studies.
Wajda et al., 2017	Individuals with neurodegenerative disease (PD, multiple sclerosis, AD, other dementia and MCI).	21	721	RCT (10), NRCT or Pre-Post (11)	Dual-task (motor task and cognitive task) interventions in individuals with NDD.	Intervention modalities for targeting cognitive-motor interference.	Results of the intervention showed that multiple task gait velocity increased by ∼0.1 m/s at posttest and was maintained after a three-week retention phase.

Abbreviations: N: Total sample size; RCT: randomized clinical trial; NRCT: non-randomized controlled trial; RPD: repeated measure designs; CT: controlled trials; PD: Parkinson disease; AD: Alzheimer disease; MCI: mild cognitive impairment; DTT: dual-task training.

The reviews were published between 2011 and 2018. A total of 133 primary studies were assessed by the systematic reviews considered in this work. There was a range varying from seven, Plummer and Iyigün (2018), to twenty-eight studies, Pichierri et al., 2011, in the systematic reviews considered. The designs of studies evaluated varied significantly, e.g., randomized controlled trials (RCT), non-randomized controlled trials (NRCT) (Agmon et al., 2014; He et al., 2018; Lauenroth et al., 2016; Law et al., 2014; Pichierri et al., 2011; Plummer and Iyigün, 2018; Wajda et al., 2017); non-blinded RCT, single-blinded RCT, double-blinded RCT, test-retest (Fritz et al., 2015); case study, case-control, two groups control, pre-post (Pichierri et al., 2011).

The reviews comprised studies evaluating individuals with brain injury, Parkinson’s disease (PD), Alzheimer’s disease (AD) (Fritz et al., 2015); stroke, traumatic brain injury, with history of falls and balance disorders, cognitive impairment, osteoporosis (Pichierri et al., 2011); with cognitive impairment or dementia (Law et al., 2014); cognitive complaints, subacute stroke, dementia, AD, cognitively healthy (Lauenroth et al., 2016); neurodegenerative diseases (NDD), including multiple sclerosis, PD, AD and mild cognitive impairment (MCI) (Wajda et al., 2017); stroke (He et al., 2018; Plummer and Iyigün, 2018) and even exclusively healthy elderly (Agmon et al., 2014; Lauenroth et al., 2016; Law et al., 2014;).

The age of participants varied shortly with half of the systematic reviews considering patients above 60 years old (Agmon et al., 2014; Law et al., 2014; Pichierri et al., 2011; Wajda et al., 2017); and the other 50 % of the studies permitted participants from 18 years old and above (Fritz et al., 2015; He et al., 2018; Lauenroth et al., 2016; Plummer and Iyigün, 2018). In terms of the outcomes, four studies focused on cognitive functions (He et al., 2018; Lauenroth et al., 2016; Law et al., 2014; Pichierri et al., 2011); and the others had primary outcomes planned as mobility performance or posture stability, besides the cognitive outcomes assessed. (Agmon et al., 2014; Fritz et al., 2015; Plummer and Iyigün, 2018; Wajda et al., 2017). Whereas all of them applied dual-task as an intervention, as it was a pre-requisite for their selection for the present analysis (Table 1).

### 3.2 Synthesis of findings

Based on our analysis of findings regarding the included reviews, two main themes were identified and developed, each addressing our overall aim to investigate the effect of dual-task training on cognition, stability or mobility of people with different clinical conditions: Improvement on mobility performance or posture stability; and beneficial effect on cognitive function (Table 1).

#### 3.2.1 Beneficial effect on cognitive function

According to He et al. (2018) and their study, dual-task mobility training was observed to prompt more improvement in single-task walking function (standardized effect size between 0.14 and 2.24), comparing with single-task mobility training. It was not reliable its effect on dual-task walking function. Cognitive-motor balance training improved single-task balance function (standardized effect size between 0.27 and 1.82), but its effect on dual-task balance ability was not analyzed. Only one study provided the beneficial effect of dual-task training on cognitive function, therefore inconclusive. Also, Lauenroth et al. (2016) findings suggests that training program from 1–3 hours weekly, for 12 weeks or more has a bigger chance to lead to noticeable improvements in cognitive performance when compared to different training schemes. Moreover, in Law et al. (2014) review, it was showed that studies with cognitively healthy populations revealed significant benefits of combined cognitive and exercise interventions on general cognitive functions, memory and functional status compared to active control group. Studies with cognitively impaired populations also showed significant improvements in general cognitive function, memory, executive functions, attention and functional status in persons with MCI and AD or dementia but lack comparison with active control groups.

#### 3.2.2 Improvement on physical functioning or performance

Agmon et al. (2014) demonstrated the potential to increase postural control, improving balance and walking ability in older adults. Fritz et al. (2015) showed that DTT improves single-task gait velocity and stride length in subjects with PD, AD and brain injury, besides the possibility of improving balance and cognition in those with PD and AD. According to Pichierri et al. (2011) and their findings, the evidence was considered limited about the effectiveness of cognitive-motor or cognitive interventions to enhance physical functioning in elderly or patients with history of traumatic brain injury. However, they point that overall, most studies comprised in their review indicated that the interventions could improve physical performance. Plummer and Iyigün (2018) showed that exercise and gait training interventions, especially involving dual-task practice, may improve dual-task gait speed after stroke, but the clinical significance is unclear. Dual-task gait speed was increased by 0.03 m/s (95 % Confidence Interval) and authors state that dual-task interventions had larger treatment effect on dual-task gait speed than those of interventions without dual-task training. Current effect size estimates lack precision due to small sample sizes of existing studies (Plummer and Iyigün, 2018). Wajda et al. (2017) found an increase of 0.1 m/s for gait velocity of patients with PD after intervention with dual-task and the improvement was maintained even after 3 week retention phase.

Table 2 summarizes the effects of DTT in each condition analyzed. Results were grouped focusing on population studied, range of follow-up length informed in the studies and implication on outcome. Cognitive function was improved with DTT in studies reported in Law et al. (2014) for healthy individuals and those with mild cognitive impairment; Fritz et al. (2015) for individuals with brain injury, Parkinson disease and Alzheimer disease, also in Lauenroth et al. (2016) for healthy individuals, with mild cognitive impairment, after stroke and with Alzheimer disease. Mobility performance showed improvement in Pichierri et al. (2011), Agmon et al. (2014), Fritz et al. (2015), Wajda et al. (2017), He et al. (2018) and Plummer and Iyigün (2018). Postural stability was enhanced in studies analyzed by Pichierri et al. (2011), Agmon et al. (2014), Fritz et al. (2015), Wajda et al. (2017) and Plummer and Iyigün (2018).

**Table 2:** Effects of DTT in different populations and outcomes.

Author	Population	Follow-up length	Cognitive function	Mobility performance	Posture stability
Pichierri et al., 2011	MCI, stroke and BI	2−12 weeks	X	↑	↑
Agmon et al., 2014	Healthy	2−4 weeks	X	↑	↑
Law et al., 2014	Healthy and MCI	2−60 months	↑	N/A	N/A
Fritz et al., 2015	BI, PD and AD.	12 weeks	↑	↑	↑
Lauenroth et al., 2016	Healthy, MCI, stroke and AD.	5 years	↑	N/A	N/A
Wajda et al., 2017	PD, MS, AD and MCI	1−12 months	N/A	↑	↑
He et al., 2018	Stroke	2 weeks	X	↑	N/A
Plummer and Iyigün, 2018	Stroke	1−6 months	N/A	↑	↑

Abbreviations: ↑ = Improve in most studies that analyzed this outcome; X = No effect in most studies that analyzed this outcome; N/A = Not analyzed; BI = Brain injury; PD = Parkinson disease; AD = Alzheimer disease; MCI = Mild cognitive impairment; MS = Multiple sclerosis.

According to provided data, DTT protocols may improve cognition and motor skills. However, different protocols bring different results for distinct populations. Longer follow-ups were reported in studies with positive findings for cognitive function, with at least two months length (Law et al., 2014). For motor skills such as mobility performance or postural stability/balance, shorter follow-ups were informed: 2 weeks minimum (Pichierri et al., 2011; Agmon et al., 2014; He et al., 2018). Apart from multiple sclerosis (cognitive function was not analyzed in Wajda et al. (2017)), all conditions explored showed some improvement with DTT in at least one of the studies. For example, patients after stroke did not improve cognition for Pichierri et al. (2011) and He et al. (2018) protocols, but they showed improvement in Lauenroth et al. (2016).

Table 3 provides the AMSTAR qualification of evidence for the systematic reviews included. In terms of quality of evidence, about 62.5 % were rated as being of “low” quality (Agmon et al., 2014; Fritz et al., 2015; Pichierri et al., 2011; Law et al., 2014; Wajda et al., 2017). 37.5 % of the reviews were graded as of “moderate” quality (He et al., 2018; Lauenroth et al., 2016; Plummer and Iyigün, 2018). While none of the systematic reviews included achieved a “high” quality grade.

**Table 3:** AMSTAR classification of systematic reviews included.

Criteria	Agmon et al., 2014	Fritz et al., 2015	He et al., 2018	Lauenroth et al., 2016	Law et al., 2014	Pichierri et al., 2011	Plummer and Iyigün, 2018	Wajda et al., 2017
1. was a “priori” design provided?	Yes	Yes	Yes	Yes	Yes	No	No	Yes
2. was there duplicate study selection and data extraction?	Yes	No	No	Yes	Yes	Yes	Yes	No
3. was a comprehensive literature performed?	No	No	Yes	No	No	No	Yes	Yes
4. was the status of publication used as an inclusion criterion?	No	No	No	No	No	No	No	No
5. was a list of studies (included and excluded) provided?	No	No	No	No	No	No	Yes	No
6. were the characteristics of the included studies provided?	No	Yes	Yes	Yes	Yes	Yes	No	Yes
7. was the scientific quality of the included studies assessed and documented?	No	Yes	Yes	Yes	No	Yes	Yes	Yes
8. was the scientific quality of the included studies used appropriately in formulating conclusions?	No	No	No	No	No	No	No	No
9. were the methods used to combine the findings of studies appropriate?	Yes	No	No	Yes	No	No	Yes	No
10. was the likelihood of publication bias assessed?	No	No	Yes	Yes	No	No	Yes	No
11. was the conflict of interest stated?	Yes	Yes	Yes	Yes	Yes	Yes	No	Yes
Total	4	4	6	7	4	4	6	5
Score	**Low**	**Low**	**Moderate**	**Moderate**	**Low**	**Low**	**Moderate**	**Low**
Details	No Meta-analysis	No Meta-analysis	No Meta-analysis	No Meta-analysis	No Meta-analysis	No Meta-analysis	Meta-analysis	No Meta-analysis

Only one of the systematic reviews has a meta-analysis, Plummer and Iyigün (2018), hence the Cohen effect size was used with its results. The result was of “no effect”. Despite bringing results in terms of significance of DTT on cognition, the other seven reviews’ data are not available for classification of overall effect size.

## 4 Discussion

This overview examined the effect of dual-task training on cognitive and motor functions of people with different clinical conditions. Eight studies were identified in the present review, seven included a population with some degree of morbidity in terms of cognitive function, three studies included a healthy population and one study addressed exclusively healthy elderly people. Among the conditions covered, there was neurodegenerative diseases like Parkinson’s disease, Alzheimer’s disease, multiple sclerosis, dementia other than AD, mild cognitive impairment, individuals with brain injury and stroke survivors. Also, there were participants with conditions such as history of falls, balance disorders and even osteoporosis. The reviews were published within the past 9 years (2011–2018) and the research and sites of data collection were from a great variety of countries. This suggests that the issue of investigation of cognitive parameters related to practice of dual-task training is not restricted to developed countries and it is worldwide.

The findings of the present work suggest an improvement in cognitive or motor function for those who experienced dual-task training. Three studies reported improvement in cognitive functions (Fritz et al., 2015; Lauenroth et al., 2016; Law et al., 2014) and five showed improvement in motor functions (Agmon et al., 2014; Fritz et al., 2015; Pichierri et al., 2011; Plummer and Iyigün, 2018; Wajda et al., 2017). For the improvement in cognition and motor function reported by He et al. (2018), it was found significant results in three of his thirteen studies encompassed and for the following tests: Stroop test (medium effect size) and Trunk Impairment Scale (large effect size). The DTT modalities used by the studies involved were walking-cognitive dual-task training, balance plus motor or cognitive tasks training. Lauenroth et al. (2016) found that successful DTT should include cardiovascular training and strength training sessions plus attention or executive function / working memory practice. Those items were reported to be desirable to yield a positive influence on cognitive performance. Finally, the authors recommend an increasing level of difficulty, with caution not to be excessive. Law et al. (2014) found important improvement in cognition of cognitively healthy population subjected to DTT. The authors reported that their participants had to be sufficiently challenged to reach the effects, which allows an understanding of a need to individualize the test according to each participant. Also, the intervention period for successful outcome with cognitively healthy individuals was reported to be smaller than for those with MCI.

When it comes to assess the effect size among studies it is uncertain due to the great variety of different methods for data analysis and different conditions reported in the studies covered. The results varied within a big range among reviews, having results showing significant effect at one side and studies lacking data for calculation and analysis on the other side. Pichierri et al. (2011) and Lauenroth et al. (2016) had the biggest number of participants in the studies reported in their reviews when compared to the other reviews in this meta-synthesis, reaching more than one thousand individuals. However, despite the robustness expected for their work when considered the n total, their results point to some limited conclusion with data not available for calculation and comparison of effect size. More recent reviews like the ones of Plummer and Iyigün (2018) and Wajda et al. (2017) have smaller numbers of individuals, 125 and 721, respectively. However, those recent studies bring more conclusive results, despite the important heterogeneity of their individuals, e.g., various degenerative disorders in Wajda et al. (2017).

The results found show a perspective that, if neglected, will have worrying implications for the large-scale health scenario. This is because cognitive impairment in all its variety of presentations are directly related to population aging. Also, those morbidities were fairly compared to cardiovascular diseases, the most frequent cause of morbidity and mortality in the world (Hurd et al., 2013). Therefore, the findings of the present study strengthen the policies and recommendations of different health organizations, such as the World Health Organization (WHO), regarding the regular practice of physical activity, perhaps suggesting a new habit, regular and supervised practice of dual-task training.

Findings herein obtained may contribute to improve the current health context of elderly, as they reveal to professionals in the field, institutions and people at different levels of decision, that the subject in question needs more attention. The study allows the use of information and knowledge built for the practice of planning actions, setting priorities, allocating capital, evaluating existing projects and programs, as well as for the construction and execution of actions aimed at health maintenance.

It is possible to contribute to the proposition of public health policies, expressed as guidelines, indicators, determinants and conditions, in the sense of allowing reflection and discussion to primary health care professionals for the development of actions in the context of long-stay institutions, in addition to health and rehabilitation facilities. Reinforcing the improvement of its actions, especially for the planning and execution of practices focused on a balanced exercise practices associated with the practice of cognitive activities as being the path to a healthy lifestyle. The rehabilitation facilities and long-stay institutions are important spaces for access to the affected public and it is necessary to stimulate institution’s health promotion programs to reduce health risk behaviors, as well as encourage protective behaviors, such as supervised DTT.

These results should be considered with caution, due to the imprecision in establishing a direct causal relationship, since it is a broad review study. Another limitation is due to the use of different reviews counting with different populations. It should be noted that this is a study conducted with a representative sample of individuals, in which results were obtained capable of revealing associations and conclusions relevant to the research objective.

New systematic reviews strictly following the AMSTAR and PRISMA guidelines are needed. This requirement is based on the low methodological quality evidenced in the studies inserted. The improvement demand could be associated with multiple factors, like heterogeneity of the population and experimental designs applied for instance. Hence, core directions for further studies of DTT on cognition and motor function ought to include the following: precise requirements and standards for populations to be studied and rigorous compliance with methodological guidelines.

There was investigated a substantial number of studies, however with important heterogeneity, hence not allowing for further critical analysis of the results for separate groups of populations with cognitive impairment. Moreover, about the search, the terms were limited to title, abstract and keywords to limit the magnitude of the search yield to a manageable size for a limited number of researches and exclusively citations published in English were entered. The restrictions mentioned certainly limit the coverage of all aspects related to the topic researched. Further, we suggest that more randomized blinded controlled trials are made within the topic, covering individual conditions and provide more detailed data and effect sizes.

## 5 Conclusion

Findings from this overview show that people with different clinical conditions could benefit from dual-task training, despite the great variation within effect size among the studies presented. Although many effects are highlighted regarding physical functions such as mobility, gait and balance, benefits also encompass general cognitive functions. Healthy patients were evaluated, and among the clinical conditions, there were individuals with MCI, dementia, AD, PD, stroke and multiple sclerosis. Most individuals were 60 years old and above, with few adults under that age. On the other hand, there was a lack of evidence comparing effect sizes in some studies considered.

General cognitive functions, memory, attention, and functional status were assessed and improved for cognitively healthy populations, as well as cognitively impaired like MCI and AD subjected to DTT. Also, patients with stroke pathology benefited from DTT, with a regular weekly training scheme for 12 weeks or more. Studies with moderate level of evidence supported these findings. All individuals benefited in various grades from the intervention when considered motor function like postural stability or mobility, with studies varying from low to moderate level of evidence.

## Conflicts of interest

The authors have no conflicts of interest in this study.
